# Hydrogen Sulfide Affects Radical Formation in the Hippocampus of LPS Treated Rats and the Effect of Antipsychotics on Hydrogen Sulfide Forming Enzymes in Human Cell Lines

**DOI:** 10.3389/fpsyt.2018.00501

**Published:** 2018-10-16

**Authors:** Olaf Sommer, Rosana L. Aug, Andreas J. Schmidt, Philip Heiser, Eberhard Schulz, Helmut Vedder, Hans-Willi Clement

**Affiliations:** ^1^Department of Child and Adolescent Psychiatry, Psychotherapy, and Psychosomatics, Albert-Ludwigs-University Freiburg, Freiburg, Germany; ^2^Department of Psychiatry and Psychotherapy, Philipps-University, Marburg, Germany

**Keywords:** schizophrenia, electron spin resonance spectroscopy (ESR), reactive oxygen species (ROS), hydrogensulfide (H_2_S), lipopolysaccharide (LPS)

## Abstract

**Objectives:** Psychiatric disorders, such as schizophrenia and other neuroinflammatory diseases are accompanied by an increase in the oxidative stress and changes in the immune system and in the metabolic, hormonal and neurological components of the central nervous system (CNS). Hydrogen sulfide (H_2_S) is a gaseous molecule that is endogenously produced in the peripheral and central nervous system through cysteine by the following major H_2_S producing enzymes in the brain: cystathionine-γlyase (CSE), cystathionine ß-synthase (CBS) and 3-mercaptopyruvate sulfurtransferase (MPST). The physiological effects of H_2_S are broad, with antioxidative properties being a major role in the body. The aims of our investigation were to analyze the central nervous antioxidant, metabolic and neuronal effects in the hippocampus of the rat after inflammatory peripheral lipopolysaccharide (LPS) treatment; and to examine the effects of antipsychotics on the expression of these enzymes in human cell lines.

**Material and Methods:** Male Lewis rats (250 g) received an i.p. LPS injection (1 mg/kg) 24 h before microdialysis experiments. Conscious rats were infused via these probes (1.5 μl/min) with a radical scavenger 1-hydroxy-3-methoxycarbonyl-2,2,5,5-tetramethylpyrrolidine (CMH) in Krebs-Ringer solution. Sodiumhydrogensulfide (NaHS, 10 μg/min) was infused after a 2- h baseline for 1 h. Corticosterone, glutamate, glucose and lactate were measured by Elisa. Reactive oxygen species (ROS) were detected by electron spin resonance spectroscopy (ESR). The impact of the antipsychotics haloperidol, clozapine, olanzapine and risperidone on the expression of genes encoding the key enzymes of H_2_S synthesis was studied at the human neuroblastoma SH-SY5Y and monocytic U-937 cell lines. The cells were incubated for 24 h with 30 μM antipsychotic following which mRNA levels were measured by polymerase chain reaction.

**Results:** Microdialysate glucose and lactate levels dramatically increased in the hippocampus of LPS untreated rats by local application of NaHS. By contrast, in the LPS pretreated rats, there was no effect of NaHS infusion on glucose but a further significant increase in microdialysate lactate was found. It was LPS pretreatment alone that particularly enhanced lactate levels. There was a marked increase in hippocampal microdialysate glutamate levels after local NaHS infusion in LPS untreated animals. In LPS treated rats, no change was observed by NaHS, but LPS itself had the strongest effect on microdialysate glutamate levels. Microdialysate corticosterone levels were reduced by NaHS in both LPS pretreated and untreated rats. The formation of free radicals in the hippocampus significantly reduced in LPS pretreated rats, while in LPS untreated rats a significant increase was observed after NaHS infusion. In human SH-SY5Y and U-937 cells, all three major enzymes of H_2_S-Synthesis, namely cystathionine-γ-lyase, cystathione ß-synthase and 3-mercaptopyruvate sulfurtransferase, could be detected by PCR. The antipsychotics haloperidol, clozapine, olanzapine and risperidone affected all three enzymes in different ways; with haloperidol and risperidone showing major effects that led to reductions in CBS or CSE expression.

**Discussion:** The local application of NaHS in the hippocampus of the rat strongly affected glucose, lactate and glutamate release. Contrastingly, in LPS pretreated rats, a decreased radical formation was the only effect found. H_2_S synthetizing enzymes may be involved in antipsychotic mechanisms, although no clear common mechanism could be found.

## Introduction

Psychiatric disorders, such as schizophrenia and other neuroinflammatory diseases are accompanied by an increased oxidative stress, changes in the immune system and in metabolic, hormonal and neurological components of the central nervous system (1–22). More and more evidence suggests various dysregulations of the hypothalamus-pituitary-adrenal (HPA)-Axis in the course of numerous mental disorders, such as affective disorders (23–25), and schizophrenia (18–21).

The response to antipsychotics in schizophrenia shows a high variability. There are several patient cases that are resistant to clozapine, which is called “ultra-resistance to treatments in schizophrenia” (UTRS). Peripheral inflammations are associated with UTRS (26).

Hydrogen sulfide (H_2_S) is a gaseous molecule that is endogenously and enzymatically produced in the peripheral and central nervous system by three major H_2_S producing enzymes: cystathionine-γ-lyase (CSE) (27), cystathionine ß-synthase (CBS) (28) and 3-mercaptopyruvate sulfurtransferase (MPST) (29, 30); besides other mechanisms (31). High endogenous concentrations of H_2_S were found in the hippocampus and cerebellum which are parts of the human brain where CBS seems to be the most important enzyme for the synthesis (32, 33).

H_2_S is known to regulate a multitude of physiological and pathophysiological functions in the vascular, immune and nervous system [for review see (34, 35)]. There is evidence for a role of H_2_S in neurodegeneration (36), but also radical scavenging effects regarding nitric oxide (37) or glutamate mediated oxidative stress (38). Other authors report a role for H_2_S in Alzheimer's disease (39) or Parkinson's disease (40).

Promising studies have demonstrated the therapeutic effect of H_2_S donation in various disease models of cancer, inflammation or neuroinflammation (41, 42). Additionally, the pharmacological inhibition of H_2_S production (43–47) or the genetic deficiency of H_2_S producing enzymes (48–50) results in beneficial effects.

Xiong et al. could show that the H_2_S levels of patients with schizophrenia are significantly low. H_2_S is a regulator for the N-methyl-D-aspartate receptor (NMDAR) function and low H_2_S levels can cause a hypofunction of NMDAR. Given that a hypofunction of the NMDAR receptor is related to the pathogenesis of this disorder, H_2_S levels in the liquor are also likely to have an effect on the pathophysiology of this disorder (51).

The aims of our investigation were to analyze antioxidant, metabolic, hormonal and neuronal changes of NaHS application in the hippocampus in naive and LPS pretreated rats. Furthermore, we aim to show the effects of different antipsychotics on the expression of several enzymes related with the H_2_S synthesis in a neuronal and an immunological human cell lines. Especially the role of exogenous H_2_S as a possible therapeutic mediator in inflammatory and neurodegenerative diseases will be analyzed.

## Materials and methods

### Animals

Animal experiments were carried out at the Department of Surgical Research, University Hospital, Freiburg, Germany. All procedures were performed in accordance with the German animal protection law, FELASA, the national animal welfare body, GV-SOLAS and the NIH guide for the care and use of laboratory animals; and were approved by the animal welfare committee of the University of Freiburg (AZ: G-10/44). Male Lewis rats (250–350 g; Charles River, Germany) were housed at a temperature of 21° C in plastic cages with lights turned on from 06:00 to 19:00 h and with free access to food and water. Experiments were conducted during the light phase after at least 1 week of adaptation.

### *In vivo* microdialysis experimental design

CMA/12 microdialysis probes were implanted under isoflurane anesthesia (3%) using stereotaxic coordinates according to (52) A: +5.2 mm; L: +2.0 mm; V: −4.4 mm from Cortex top (53). The microdialysis experiments started after awakening using the CMA freely moving system in groups of 8 animals. Microdialysis samples were collected every 30 min for a period of 4 h at a constant flow rate of 1.5 μL/min (Krebs-Ringer). Microdialysis samples were collected and stored at −20°C until analysis. Electron spin resonance (ESR) measurements were performed immediately. The same effluents were used to measure Corticosterone (Enzyme immunoassay, IBL, Hamburg, Germany), Glucose, Lactate and Glutamate (Colorimetric Assays, BioCat, Heidelberg, Germany).

To detect central nervous protective effects of hydrogen sulfide, a series of rats received an intraperitoneal injection of 1 mg/kg body weight Lipopolysaccharide from *E. coli* (LPS; Sigma Aldrich Chemicals, Steinheim, Germany). The injections were made 24 h before microdialysis probes were implanted. NaHS was infused intrahippocampal via the microdialysis cannula at a flowrate and dose of 10 μg/min 2 h after beginning of sampling. ROS, Glutamate, Corticosterone, Lactate and Glucose-Uptake (difference between glucose inflow concentration and outflow concentration) were measured.

### Histology

At the end of the experiment, one group of animals was euthanized with CO_2_. To verify probe placement, the brain was removed and stored at −20°C. Subsequently, serial coronal brain sections (thickness: 20 μm) were cut on a freezing microtome at −16°C and sections were stained with a 0.5% cresyl violet solution.

### Chemicals

Krebs-Ringer's solution was obtained from Delta-Select, Pfullingen, Germany; LPS (Lipopolysaccharides from Escherichia coli, Serotype 0127:B8 purified by trichloroacetic acid extraction) from Sigma, Steinheim, Germany; 1-hydroxy-3-methoxycarbonyl-2,2,5,5-tetramethylpyrrolidine (CMH) from Noxygen, Elzach, Germany; Isofluorane from Abbott, Wiesbaden, Germany; Primers were obtained from MWG Biotech, Ebersberg, Germany.

### ROS measurements

Hippocampal detection of reactive oxygen species is based on the reaction of CMH and ROS as reactant in dialysates. CMH solution (1 mg/ml Krebs-Ringer) was prepared fresh every day. The spin probe CMH was applied by infusion via a microdialysis cannula at a flow rate of 1.5 μl/min. The oxidation of spin probe CMH by reactive oxygen species generates stable 3-methoxycarbonyl-proxyl radicals (CM) as shown by Dikalov et al. (54, 55). Autoxidation was found to be in the range of 1–2%. The amount of CM. radical is equivalent to the formation of reactive oxygen species *in vivo*. The amount of reacted ROS was determined from the ESR amplitude according to a calibration curve using standard CM. solutions. ESR measurements were performed at room temperature using an EMX ESR spectrometer (MiniScope MS 200, Magnettech, Berlin, Germany). The ESR had the following settings: center field *g* = 2.001, sweep wide 60 G, sweep time 5 ms over 10 scans, modulation amplitude 2.4 G, microwave power 20 mW. The total spin probe concentration was measured to determine the concentration of free radicals (56).

### Cells and culture conditions

U-937 cells were maintained from the Roswell Park Memorial Institute (RPMI) 1640 medium supplemented with 10% heat-inactivated fetal calf serum (FCS) (Gibco/BRL and Seromed, Berlin, Germany), 0.5% glutamine, and 1% gentamycine at 37°C in a 5% CO_2_ atmosphere. For the studies, cells were plated at a concentration of 225,000 cells in 3 ml medium per well into six-well culture plates (Greiner, Frickenhausen, Germany).

Cells were preincubated for 24 h and then treated with the antipsychotics, dissolved in ethanol at a concentration of 30 μM. These were added to the plates at a quantity of 300 μl to each well. The same procedure was performed with the ethanol controls without antipsychotics. Antipsychotics were obtained from Sigma, Deisenhofen, Germany.

Neuroblastoma SH-SY5Y cells were cultured in heat-inactivated Roswell Park Memorial Institute medium (RPMI) (Gibco/BRL, Eggenstein, Germany) supplemented with 15% fetal calf serum (FCS) (Biochrom, Berlin, Germany), 1% penicillin-streptomycin and 1% glutamine in a 5% CO2 atmosphere. For further studies, cells were plated at a number of 225.000 cells/dish in 10 mm culture dishes. Antipsychotics and ethanol-control treatments were performed at a density of 450.000 cells/dish. The antipsychotics were dissolved in ethanol and were further diluted in culture medium. The cells were exposed to antipsychotics at 30 μM for 24 h at 37°C.

### RNA extraction

After incubation, the cells were collected from the culture dishes and total RNA was extracted by the use of Trizol® reagent in accordance with the manufacturer's instructions. The amount of extracted total RNA was quantified by established optical methods at A260/A280 (Genequant II, Pharmacia Biotech, Freiburg, Germany) and structural integrity checked by agarose-gel electrophoresis [1.5% agarose (Gibco/BRL)].

### Reverse transcriptase-polymerase chain reaction (RT-PCR)

RT-PCR was used to analyse the transcription of the GRs and the house-keeping genes glyceraldehyde-3-phosphate dehydrogenase (GAPDH). 1 mg of cellular total RNA was reverse-transcribed with 40 U of Superscript II (Gibco/BRL) and 1 mg oligo-(dT) in a volume of 20 ml following the manufacturer's protocol.

The following primer pairs were used to amplify the cDNA's:

CBS: Sense 5′-CGATGGGTACCATATGCAGAAAAGACGCCTCCTCACAAGG-3′, Antisense 5′-CGGTACCTCGAGTTACTACTGTGATTCCACTTGGAGGGTGTGCTGCC-3′. CSE: Sense 5′-GGCCTGAAGTGTGAGCTCTT-3′, Antisense 5′-TTGGGGATTTCGTTCTTCAG-3′. MPST: Sense 5′-GACCCCGCCTTCATCAAG-3′, Antisense 5′-CATGTACCACTCCACCCA-3′. GAPDH: Sense 5′-CGTCTTCACCACCATGGAGA-3′, Antisense 5′-CGGCCATCACGCCACAGTTT-3′.

Aliquots of 1 ml cDNA were amplified with a PCR cycler (Biometra Trio, Göttingen, Germany) for the enzymes and GAPDH using the primers described above with the following cycling program: denaturation for 45 s at 95°C, annealing for 60 s at 59°C, and extension for 60 s at 72°C. PCR products were analyzed for all enzymes after amplification with 28 cycles and gel electrophoresis in 1.5% agarose gels. Semi-quantitative determination was achieved by digitization of gels with a Polaroid video system (Rothaar & Schroeder, Heidelberg, Germany) and further densitometric evaluation achieved with the Gelscan 4.0 Professional Program (LTF/BioSciTec, Landau/Frankfurt, Germany).

### Quantification of mRNA by RT-PCR and densitometry

Different approaches were used to minimize variations and to ensure the reliability of the quantification procedure: first, the purity of mRNA probes was determined by measuring the optical density at A260/A280. This revealed a ratio of 1.56 ± 0.05 for all extractable probes. Second, integrity and amounts of mRNA measured were checked by gel electrophoresis. Third, relevant impurities of DNA were routinely excluded by PCR-amplification of extracted mRNA probes after omission of the RT reaction. The reliability of the further steps of quantification (PCR reaction, gel densitometry and evaluation of results) including non-saturating conditions of the PCR was determined using different amounts of RT products. This yielded a near-linear dose-product relationship in the gel electrophoresis.

### Statistical analyses

The statistical analysis of the microdialysis data was performed using Microsoft Excel 8.0 and SPSS version 9.0 (SPSS Inc., Chicago, USA). The given data comprises mean ± standard deviation (SD). For microdialysis experiments significance was assessed by unpaired Student *t*-test, one-tailed (since we had a clear hypothesis from the previous *in-vitro* studies). An α-level of *p* < 0.05 was considered significant (57). Data are presented in percent of the mean of the respective control samples and are shown as mean ± SD values. The results from at least three independent different experiments were pooled and analyzed by Mann-Whitney Rank Sum tests. A *p* < 0.05 (^*^) was accepted as a statistically significant difference. For statistical evaluations, Sigmastat (Jandel Scientific, Kerpenich, Germany) was applied (58).

## Results

**Figure 1:**
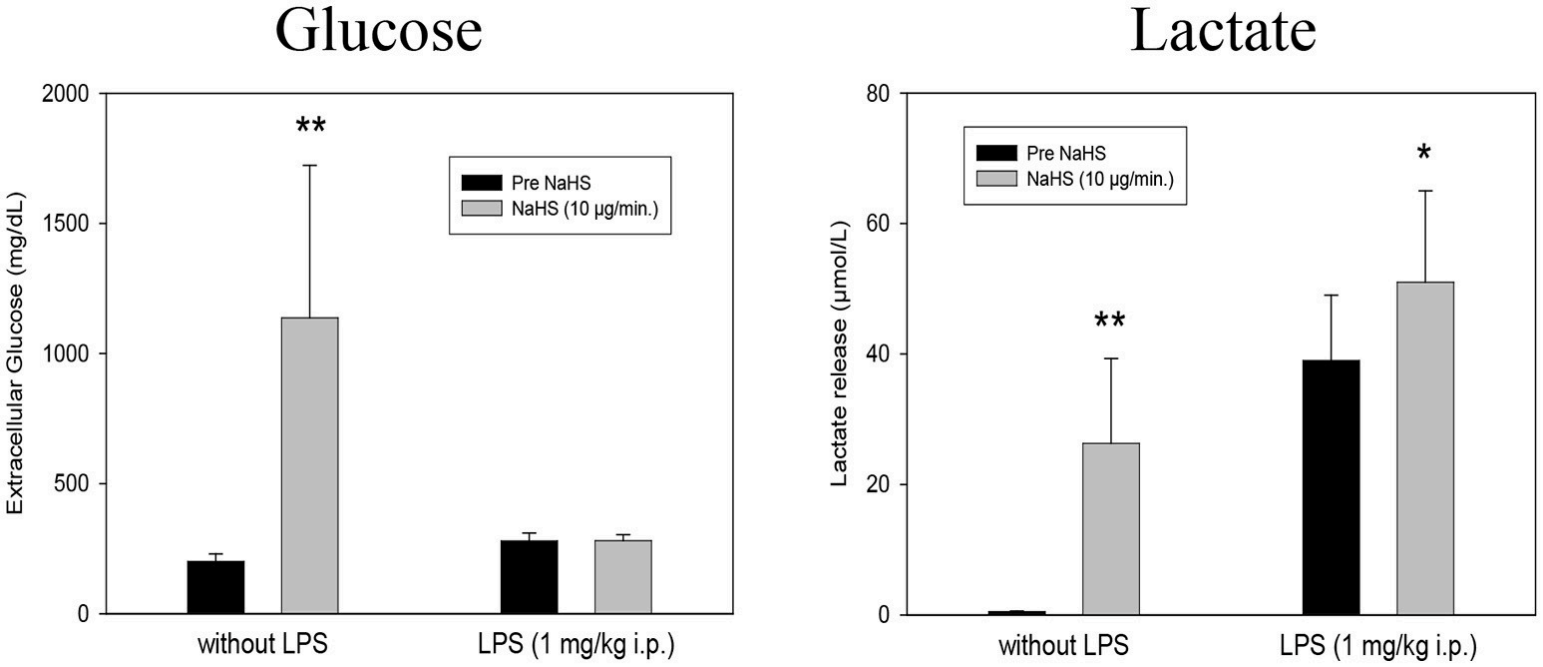
Effect of intrahippocampal NaHS infusion (10 μg/min) via CMA/12 micordialysis cannula on Glucose uptake (**Left**) and Lactate release (**Right**) with and without LPS treatment (1 mg/kg i.p., 24 h before microdialysis, LPS) as compared to control. Means ± SD, *n* = 8, *p < 0.05; **p < 0.01, Student *t*-test.

The local intrahippocampal application of NaHS via microdialysis cannula (10 μg/min. at 1.5 ml/min. for 1 h) affected different neuronal parameters. In LPS untreated rats, NaHS infusion exerted massive increases in both microdialysis glucose and lactate release in the hippocampus. These effects were not seen in LPS treated rats (1 mg/kg i.p. 24 h before microdialysis experiments), Figure 1.

**Figure 2:**
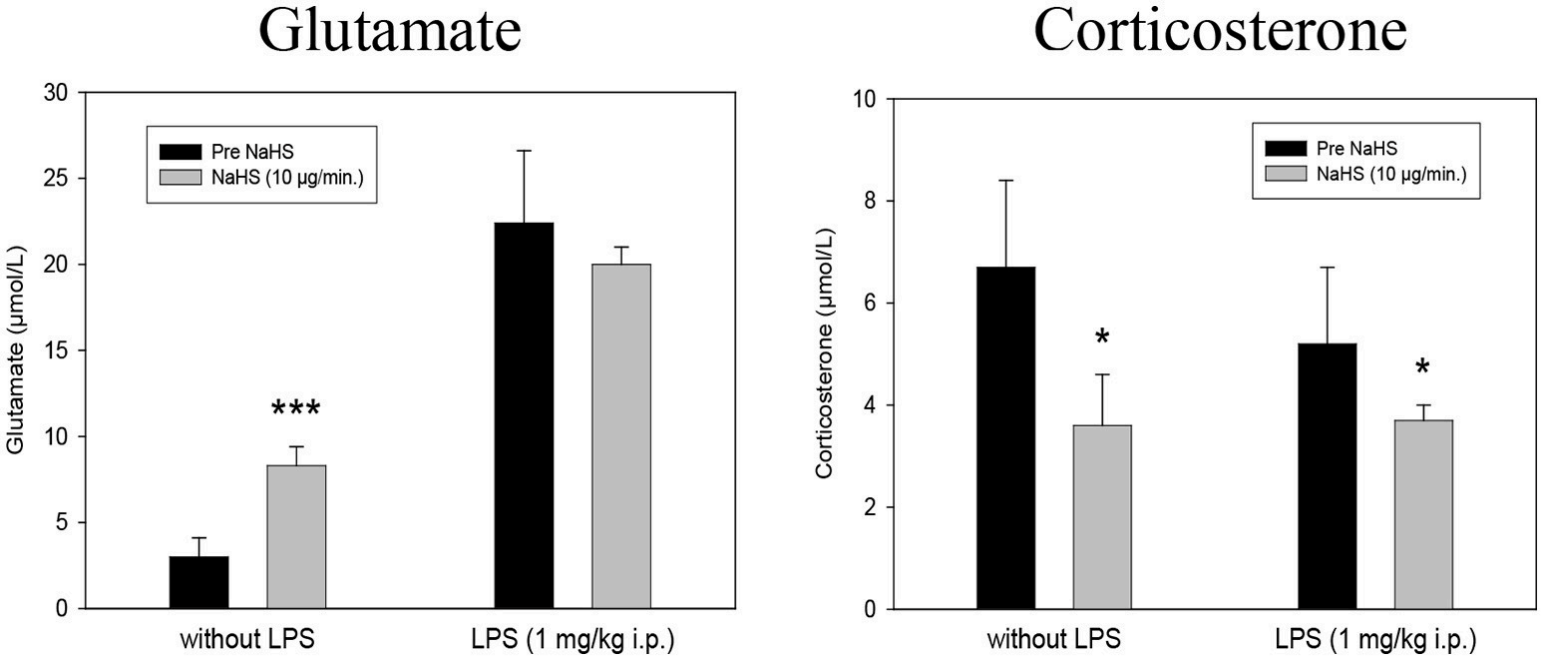
Effect of intrahippocampal NaHS infusion (10 μg/min) via CMA/12 micordialysis cannula on Glutamate release (**Left**) and Corticosterone release (**Right**) with and without LPS treatment (1 mg/kg i.p., 24 h before microdialysis, LPS) as compared to control. Means ± SD, *n* = 8, *p < 0.05; ***p < 0.001, Student *t*-test.

Microdialysis monitoring of Glutamate and Corticosterone showed a highly significant increase in Glutamate and a diminished formation of Corticosterone after 10 μg/min NaHS compared to the previous control periods. In the LPS treated rats no further increase in Glutamate could be observed. The decreasing effect of NaHS on Corticosterone was also observed in LPS treated animals (Figure 2).

**Figure 3:**
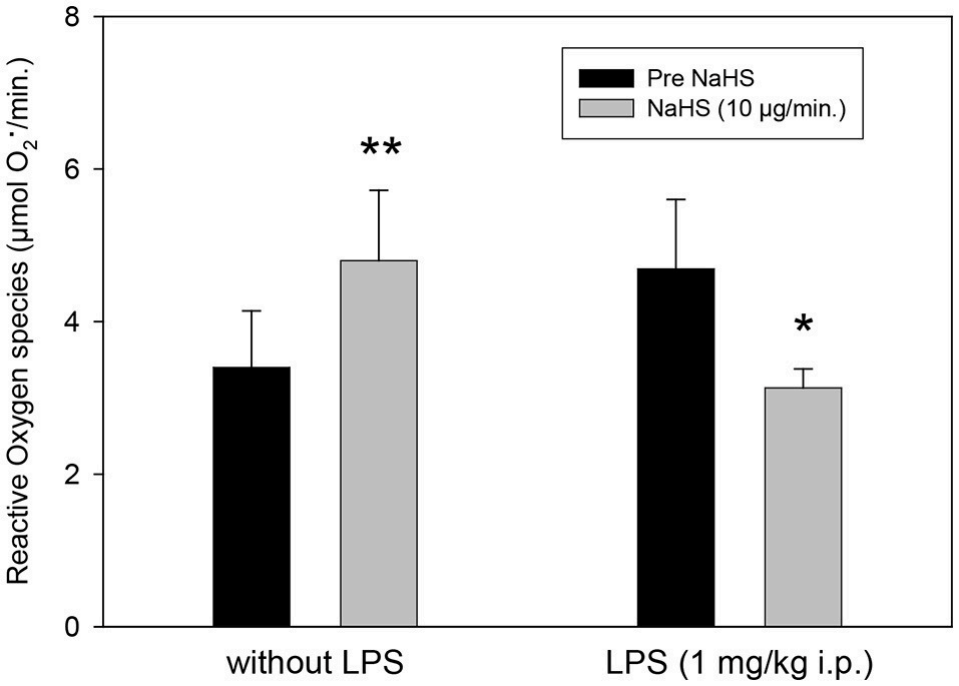
Effect of intrahippocampal NaHS infusion (10 μg/min) via CMA/12 micordialysis cannula on ROS production (as measured by CM.) with and without LPS treatment (1 mg/kg i.p., 24 h before microdialysis, LPS) as compared to control. Means ± SD, *n* = 8, *p < 0.05; **p < 0.01, Student *t*-test.

ROS are also affected significantly by NaHS (Figure 3). The infusion of NaHS, 10 μg/min, led to a significant increase in free radical formation in LPS-untreated animals. In contrary, in LPS treated animals we saw a free radical decrease of NaHS.

**Figure 4:**
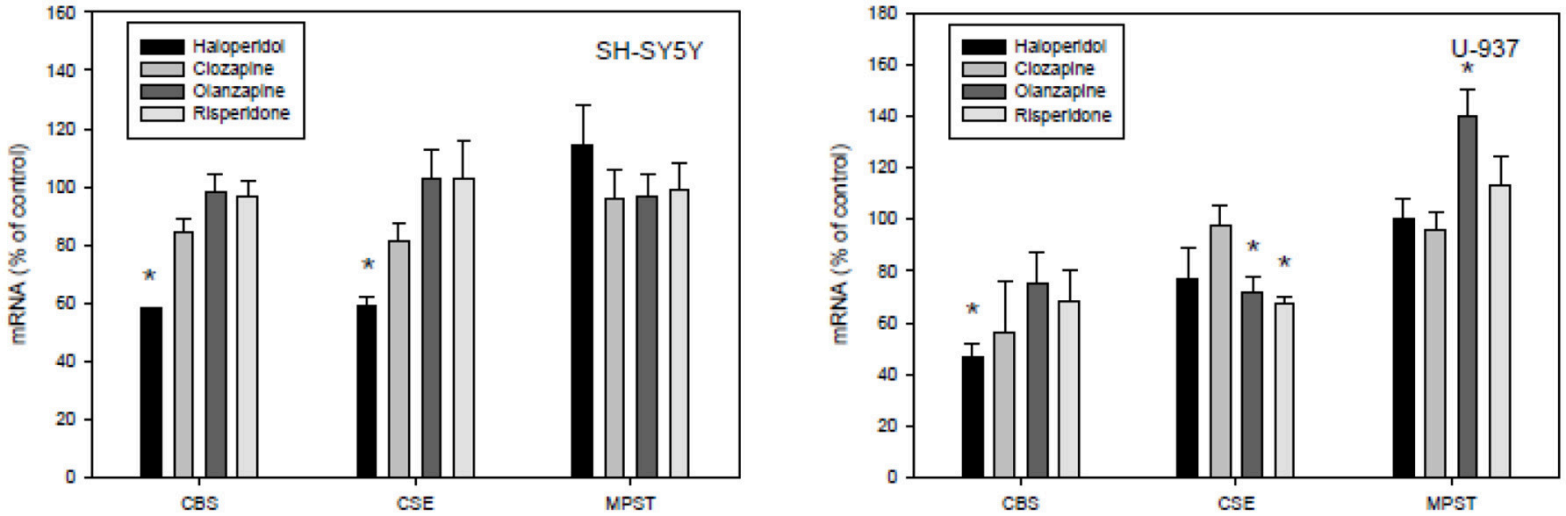
Effects of antipsychotic treatment on cystathione-y-lyase (CSE), cystathione ß-synthase (CBS), and 3-mercaptopyruvate sulfurtransferase (3MST) in human SH-SY5Y and U-937 cell lines. Means ± SD, *n* = 8, *p < 0.05; Mann-Whitney Rank Sum test.

Our present study demonstrated effects of typical (haloperidol) as well as atypical antipsychotics (clozapine, olanzapine, risperidone) on the main H_2_S synthesizing enzymes CBS, CBE, and MPST in SH-SY5Y cells, and U-937 cells (Figure 4). In essence, all measured significant effects of the used antipsychotics in the employed concentration were reductions in the expression of both enzymes CBS and CSE in the neuronal cell line. Haloperidol significantly reduced the expression of CBS and CSE in SH-SY5Y cells, but the effect of haloperidol was not significant in U-937 cells. In U-937 cells the antipsychotics olanzapine and risperidone reduced the m-RNA of CSE significantly. The expression of MPST was only affected in U-937 cells and only by olanzapine. Clozapine, the most important antipsychotic did not show any effect in both cell lines.

## Discussion

Our data indicate that H_2_S affects parameters involved in the pathophysiology of inflammatory psychiatric illnesses, such as depression and schizophrenia. Especially in normal Lewis rats, extracellular glucose, lactate release and glutamate release are highly increased in the hippocampus after local application of NaHS via microdialysis cannula. Hippocampal corticosterone levels are found to be significantly reduced under these conditions. At this concentration, an increase in free radical formation could also be observed, whereas after LPS pretreatment of animals a significant reduction could be observed. Under the conditions of LPS pretreatment the effects of NaHS on glutamate, corticosterone, glucose, and lactate release were missing. Antipsychotics seem to have individual effects on the expression of the major H_2_S-synthetizing enzymes with haloperidol exerting mostly reducing effects, while clozapine was ineffective in both SH-SY5Y and U-937 cell lines.

Hayden et al. (59) have also shown elevated blood glucose levels after exposure with H_2_S. Pichette and Gagnon summarize the literature on the effects of H_2_S and glucose regulation, indicating that mechanisms besides insulin regulation, such as glucagon-like-peptide (GLP)-1 or peptide YY are involved (60). Our data confirm findings of Lin et al. (61) that exogenous H_2_S protects cells by its glucose reducing activity.

The increase in lactate by NaHS-Application may be supported by the observation that in fish exposed to H_2_S, an increase in whole blood lactate could be observed (62). Without LPS pretreatment, our results suggest that the local application of NaHS leads to a general metabolic activation of neurons and glial cells in the hippocampus.

Our findings of the significant release of glutamate in the hippocampus of the rat induced by local NaHS application might be due to postsynaptic effects of H_2_S on the membrane potential, and thus excitability of CNS neurons, as it has been reported in dorsal raphe (63), paraventricular nucleus (64), subfornical organs (65) and dorsal root ganglion neurons (66). The effects in the dorsal raphe have been suggested to be the result of direct modulatory actions of H_2_S on calcium dependent potassium channels (63).

Significant alterations in the HPA axis activity occur in course of the schizophrenia, regarding basal cortisol secretion, probably in response to a decrease in the amount of glucocorticoid receptors (25, 67, 68). Walder et al. (69) revealed a significant positive correlation between salivary cortisol concentrations and symptoms severity. A study of Walker et al. (70) revealed that increased cortisol levels in patients who developed psychosis. Therefore, the hypothesis that HPA axis distortion is closely bound with symptoms and pathophysiology of the schizophrenia is becoming increasingly recognized. Antipsychotic medication leads to the reduction of cortisol concentrations in patients as well as in healthy controls (71, 72). Flores et al. (73) suggested that this might be responsible for their effectivity (17). Wang et al. (74) showed that inhibitors of the H_2_S producing enzymes, such as CBS or CSE, or the application of small interfering RNAs lead to mitochondrial oxidative stress and dysfunction, resulting in an even blunted corticosterone response to ACTH in adrenal glands. These effects were significantly attenuated by the treatment of H_2_S donor GYY4137. As shown by Navarra et al. (75), NaHS application is associated with the inhibition of the stimulated release of corticotropin-releasing hormone from rat hypothalamic explants.

In our experiments we found a significant reduction on free radical production in the hippocampus induced by the local NaHS application in the LPS pretreated rats. These findings support the role of H_2_S as an important antioxidative anti-inflammatory mediator. H_2_S was found to increase the antioxidative properties of cells in different ways (76). It decreases lipid peroxidation induced by homocysteine (77). H_2_S is able to directly react with reactive oxygen but its endogenous concentrations are too low to act as an important endogenous antioxidant. On the other hand H_2_S has a cytoprotective effect in brain cells by elevating the reduced glutathione (GSH) production via activation of cystine/cysteine transporters and redistribution of GSH to mitochondria (38, 78, 79). On the molecular level, several mechanisms seem to be involved in the antioxidant and neuroprotective role of H_2_S, such as the nuclear factor (NF)-κB pathway (80). H_2_S significantly reduced levels of malondialdehyde and 4-hydroxynonenal and elevated levels of superoxide dismutase and reduced glutathione in the hippocampus of streptozotocin (STZ)-induced diabetic rats (81). They also found that H_2_S alleviated depressive-like behaviors of STZ-induced diabetic rats in the forced swimming and tail suspension tests and reduced their anxiety-like behaviors in the elevated plus maze test. The results provide evidence for antidepressant-like and anxiolytic-like effects of H_2_S in STZ-induced diabetic rats and suggest that the therapeutic effects may result from inhibition of hippocampal oxidative stress (81). In HT22 neuronal cells, Kimura et al. (82) observed a cytoprotective role of H_2_S by activating ATP-dependent K+ (KATP) and Cl- channels, in addition to increasing the levels of glutathione. In LPS-treated mice the findings of (48) indicated that a deficiency in MPST does not significantly affect endotoxemia but a deficiency in CBS or CSE slightly ameliorates the outcome of LPS-induced endotoxemia *in vivo*.

In summary our data support Huang and Moore's (83) notion, that H_2_S is not only a toxic agent but also a gasotransmitter with growing therapeutical potential. Here we could demonstrate that it might be a double sided sword and that the body condition is important for the effects of H_2_S.

We here observed that the neuroleptics haloperidol, clozapine, olanzapine and risperidone have different effects on human SH-SY5Y and U-937 cell lines. Several drugs or even vitamins, such as vitamin D_3_ (84) are able to change the concentrations of H_2_S and these effects seem to be tissue dependent (35). The mechanisms through which these effects occur are still unclear. The regulation of gene expression seems to be different in respect to tissue and cell type since antipsychotics, such as haloperidol and quetiapine seem to reduce genes encoding antioxidant enzyme expression (58), the reduction of H_2_S-forming enzymes, CBS and CSE, especially by haloperidol in neuronal cell line. It cannot be excluded that the inhibition of CSE in U-937 cells by olanzapine and risperidone is combined not only with beneficial effects of these drugs but also with shared side effects. Furthermore, Fond et al. (26) recently found that ultra-resistance to treatment in schizophrenia (URTS) is independently associated with peripheral low-grade inflammation in schizophrenia patients (26).

H_2_S does not seem to be a classical neurotransmitter as specific receptors are not reported until now. Changes in cytokine production, such as IL-1 or TNF-a in the LPS model are reported (85). Although several mechanisms report that H_2_S can exert its physiological effects, such as sulfhydration or hemeprotein interactions [for review see (31)], other mechanisms, such as the influence on receptor heterocomplexes that are important in the pathophysiology of schizophrenia cannot be excluded from consideration (86, 87).

Schizophrenic Patients have olfactory impairments as was shown by Turetsky et al. (88) and show higher depolarization responses after stimulation with H_2_S indicating a special role for H_2_S in schizophrenia. Nevertheless, it is still unclear whether there is avoidance or an even beneficial effect (88).

Taken together our data confirm that hydrogen sulfide affects mechanisms involved in the pathophysiology of neuroinflammatory diseases, such as schizophrenia and depression and antipsychotic treatment might alter H_2_S-related mechanisms. We could show that H_2_S has effects on several brain metabolites and hormones, and our data confirm the idea that H_2_S can have toxic and protective properties depending on the state the body is in, here demonstrated for the case of inflammation. Nevertheless, the collected results of the study have not adequately clarified the therapeutic benefits H_2_S has for neurodegenerative diseases in brain, and the conditions and concentrations under which the beneficial properties H_2_S exceed the toxicity of this gasotransmitter. Further beneficial effects of H_2_S and related compounds might be studied in animal models of schizophrenia.

## Author contributions

All the authors meet the following criteria: made substantial contributions to the conception or design of the work; or the acquisition, analysis, or interpretation of data for the work; and drafted the work or revised it critically for important intellectual content; and approved the final the version to be published; and agreed to be accountable for all aspects of the work in ensuring that questions related to the accuracy or integrity of any part of the work are appropriately investigated and resolved. In addition OS performed the animal experiments and AS performed the cell culture experiments.

### Conflict of interest statement

The authors declare that the research was conducted in the absence of any commercial or financial relationships that could be construed as a potential conflict of interest.
